# A Hybrid De-Noising Algorithm for the Gear Transmission System Based on CEEMDAN-PE-TFPF

**DOI:** 10.3390/e20050361

**Published:** 2018-05-11

**Authors:** Lili Bai, Zhennan Han, Yanfeng Li, Shaohui Ning

**Affiliations:** 1 College of Mechanical Engineering, Taiyuan University of Technology, Taiyuan 030024, China; 2 College of Mechanical Engineering, Taiyuan University of Science and Technology, Taiyuan 030024, China

**Keywords:** CEEMDAN, permutation entropy, TFPF, de-noising

## Abstract

In order to remove noise and preserve the important features of a signal, a hybrid de-noising algorithm based on Complete Ensemble Empirical Mode Decomposition with Adaptive Noise (CEEMDAN), Permutation Entropy (PE), and Time-Frequency Peak Filtering (TFPF) is proposed. In view of the limitations of the conventional TFPF method regarding the fixed window length problem, CEEMDAN and PE are applied to compensate for this, so that the signal is balanced with respect to both noise suppression and signal fidelity. First, the Intrinsic Mode Functions (IMFs) of the original spectra are obtained using the CEEMDAN algorithm, and the PE value of each IMF is calculated to classify whether the IMF requires filtering, then, for different IMFs, we select different window lengths to filter them using TFPF; finally, the signal is reconstructed as the sum of the filtered and residual IMFs. The filtering results of a simulated and an actual gearbox vibration signal verify that the de-noising results of CEEMDAN-PE-TFPF outperforms other signal de-noising methods, and the proposed method can reveal fault characteristic information effectively.

## 1. Introduction

The gear transmission system is commonly viewed as a crucial component of mechanical systems, due to its direct relation to the running state of the entire mechanical equipment [1,2]. Therefore, the vibration signal of gear transmission is an important indicator for measuring the stability and evaluating the health condition of running equipment. Generally speaking, vibration signals contain abundant noise, which can affect fault diagnosis accuracy [3]. In addition, many scholars have developed effective and practical signal de-noising methods [4,5], and many excellent achievements have been reported [6,7].

Moreover, most of the signal processing methods for the removal of noise have been classified into the following three categories: de-noising in the time-domain (e.g., time or space), de-noising in the frequency-domain (e.g., Fourier transform or FFT), and de-noising in the time-frequency domain (e.g., WT or EMD) [8,9,10]. Time-frequency analysis describes the characteristics of measurement signals as a two-dimensional function of both time and frequency. At present, the wavelet transform can be used to extract local features of vibration signals in both time and frequency domains [11], which is the reason why it has been widely used in rotating machinery fault diagnosis [12]. The main advantage of the wavelet transform is its excellent time-frequency localization; however, the disadvantage is that its application requires the selection of an appropriate wavelet base function and determination of the specific frequency bands with fault information.

Empirical mode decomposition (EMD) is an adaptive signal processing method which decomposes time series to Intrinsic Mode Functions (IMFs) and a residue [13]. However, it suffers from many deficiencies, such as end effects, mode mixing [14,15,16], and so on. As an extension of EMD, ensemble empirical mode decomposition (EEMD) decomposes a signal by adding white Gaussian noise and considering the average result as the true final results [17,18,19]. Even though the addition of white Gaussian noise may deal with the problem of mode mixing to some extent, the IMFs produced by EEMD often contain residual noise. Recently, the method of Complete Ensemble Empirical Mode Decomposition with Adaptive Noise (CEEMDAN) was proposed by Torres et al. [20] which eliminates the remaining noise and has been therefore widely adopted.

There are many other excellent de-noising methods in the time-frequency domain. For example, the Time-Frequency Peak Filtering (TFPF) technique is an effective random noise reduction method and has been effectively applied to random noise reduction [21,22,23]. However, there is an inherent trade-off in TFPF, that is, when a short window length is used, the amplitude of the valid signal is well preserved, but the effect of random noise reduction is poor, whereas when a long window length is applied, random noise is suppressed but the amplitude of the signal is considerably attenuated.

Moreover, some hybrid filtering methods have been proposed, and experimental results show that the performance of hybrid filters is in general superior, due to the advantages provided by their combined approach [24]. Therefore, a fusion de-noising algorithm combining CEEMDAN with TFPF is expected to be an effective method. However, various IMFs are obtained through CEEMDAN, and when each IMF is denoised through TFPF, the computational cost will be increased. To simplify the computational process, Bandt and Pompe proposed a detection method of time series randomness, namely, permutation entropy (PE) [25,26,27]. PE can be used to evaluate random noise in the signal sequence quantitatively. Therefore, the values of PE are utilized so that only IMFs with more noise are chosen for denoising, which reduces the calculation amount and shortens the calculation time.

A new hybrid de-noising method is proposed in this paper. Firstly, the original vibration signal is decomposed into a series of IMFs, and in the process of decomposition, due to the shortcomings of the EMD and EEMD algorithms, a better improved algorithm—CEEMDAN—is utilized. Then the noise reduction of each IMF is carried out. In order to reduce the computation time and amount of computation, the PE as the threshold between noisy IMFs and noise-free IMFs is introduced. By calculating the PE of each IMF, the IMF components with more noise are selected. Then the selected IMF components are de-noised by time-frequency peak filtering with different window lengths. Long windows with more noise and short windows with less noise are filtered respectively. Finally, the final reconstructed signal is obtained by combining the filtered components with the remaining components. Moreover, the performance of the proposed method is evaluated using the Signal Noise Ratio (SNR) and Mean Square Error (MSE). In addition, the fault characteristics of gears in noisy background are extracted effectively using cyclic autocorrelation spectrum analysis [28]. The reliability and superiority of the algorithm are proved by the verification of simulate signal and experimental signal, which can not only retain the effective signal to the maximum extent, but also outperform than others in noise reduction.

The remainder of this paper is organized as follows: the forthcoming section briefly introduces the theory of CEEMDAN-PE-TFPF. The section after that focuses on a simulation example of the method, while the subsequent section is devoted to the experimental demonstration of the proposed method. Conclusions are drawn in the last section.

## 2. Methodology

### 2.1. CEEMDAN

EMD, which was proposed by Huang as a data driven and fully adaptive method, is well suited for non-stationary and nonlinear vibration signals. As a novel time-frequency analysis method, EMD can decompose complicated signals into several IMFs based on the local characteristic time scale of the signal. The IMFs carry the detailed information of the signal. For noisy signals, the EMD can decompose the noisy signals into noisy signal modes and noise-less signal modes. The main task based on EMD filtering is to identify the two mode class. In EMD decomposition, however, mode mixing—in which oscillations of different amplitudes are found in a mode, or similar oscillations are encountered in different modes—often occurs. This phenomenon prevents the complete extraction of the signal’s information. To overcome this problem, Wu and Huang [29] introduced the EEMD, which is a method based on the EMD algorithm. In this method, an IMF is defined as an ensemble average of corresponding IMFs, which were decomposed from the original signal with white Gaussian noise added. This method avoids mode mixing to a certain extent. However, the EEMD algorithm leads to creation of some additional problems, such as that the added white noise is not eliminated fully, and the additional modes may be produced because of the interaction between the original signal and the white noise.

In order to overcome all of the above problems, the complete EEMD with adaptive noise (CEEMDAN) method was introduced. In this algorithm, instead of the white Gaussian noise, specific noise is added at each stage of the decomposition. Then, when a unique residue is obtained, the true IMF is defined as the difference between the current residue and the average of its local means. Hence, the problems produced by EEMD are alleviated and the CEEMDAN method, which has half the number of iterations of the EEMD method, accurately achieves signal decomposition.

**Figure 1:**
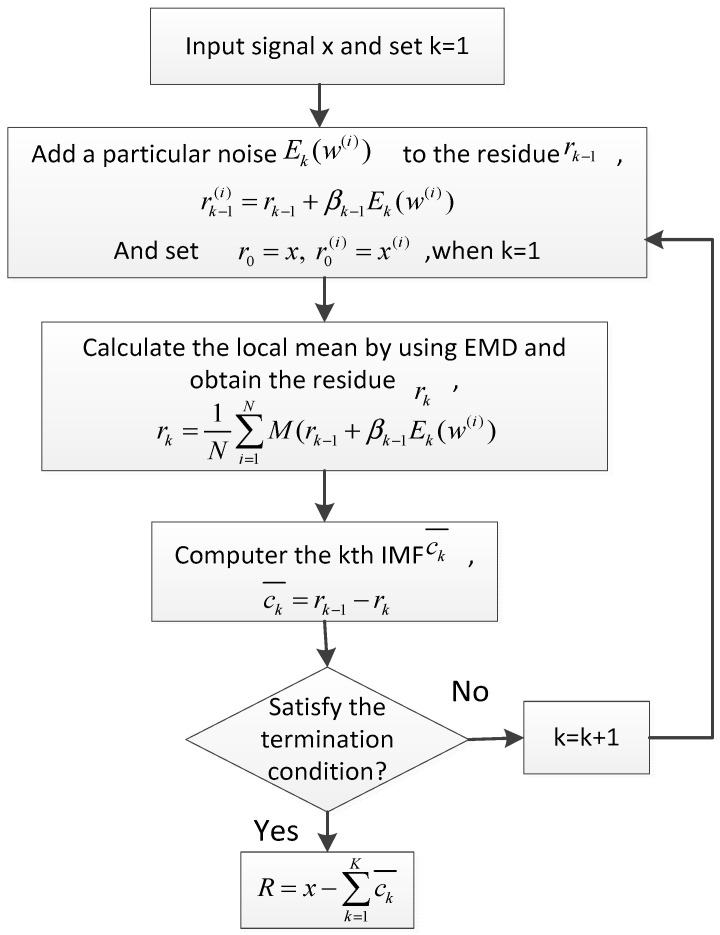
Flow chart of the CEEMDAN algorithm.

The principles of decomposition using CEEMDAN are described as follows [30] and a flow chart of the CEEMDAN algorithm is shown in Figure 1.

(1) Add $E_{1}(w^{\left(i\right)})$ to the original signal *x*:

(1) $$x^{\left(i\right)}=x+\beta_{0}E_{1}(w^{\left(i\right)})$$

where, $E_{k}(w^{\left(i\right)})$ means the *k*-th IMF of the white Gaussian noise decomposed by EMD; $E_{k}(\cdot )$ is the operator which produces the kth mode decomposed by EMD, $w^{\left(i\right)}$ indicates the *i*-th added white Gaussian noise with zero mean and unit variance, and the coefficients $\beta_{k}=\varepsilon_{0}\mathrm{std}(r_{k})$ represent the selection of the SNR at each stage.

(2) Calculate the local means of $x^{\left(i\right)}$ by using EMD and obtain the first residue $r_{1}$:

(2) $$r_{1}=\frac{1}{N}\sum_{i=1}^{N}M(x^{\left(i\right)})$$

where, N is the total ensemble number, and M(⋅) is the operator which produces the local mean of the signal, and there exists a relation that $E_{1}(x)=x-M(x)$.

(3) Compute the first IMF $\bar{c_{1}}$:

(3) $$\bar{c_{1}}=x-r_{1}$$

(4) Decompose $r_{1}+\beta_{1}E_{2}(w^{\left(i\right)})$ and define the second IMF $\bar{c_{2}}$:

(4) $$\bar{c_{2}}=r_{1}-r_{2}=r_{1}-\frac{1}{N}\sum_{i=1}^{N}M(r_{1}+\beta_{1}E_{2}(w^{\left(i\right)}))$$

(5) Estimate the *k*-th residue $r_{k}$, for *k* = 3, 4, …, *K*:

(5) $$r_{k}=\frac{1}{N}\sum_{i=1}^{N}M(r_{k-1}+\beta_{k-1}E_{k}(w^{\left(i\right)})$$

(6) Obtain the *k*-th IMF $\bar{c_{k}}$:

(6) $$\bar{c_{k}}=r_{k-1}-r_{k}$$

(7) Repeat steps 5 to 6 until the residual mode R satisfy the termination condition:

(7) $$R=x-\sum_{k=1}^{K}\bar{c_{k}}$$

Therefore, the signal x can be expressed as:

(8) $$x=\sum_{k=1}^{K}\bar{c_{k}}+R$$

### 2.2. Permutation Entropy

Entropy is used to describe the irregular and complex evolution of time series. By comparing the transformation situation of some features of signal entropy, changes in the signal’s composition can be distinguished directly [31]. The IMF components decomposed by CEEMDAN contain the local features of original signal and time scale information with different features. Permutation entropy (PE) [25], as a measure of time series complexity, is highly sensitive to time series, and it is commonly used to distinguish complex structures from white noise in a time series. The procedure for calculating PE has been established and will be briefly summarized here.

Considering the time series {x(i), i=1,2,…,N}, it is reconstructed in phase space:

(9) $$\left[\begin{array}{c} X(1)=\{x(1),x(1+\tau ),\ldots ,x(1+(m-1)\tau )\}\\ \vdots \\ X(j)=\{x(j),x(j+\tau ),\ldots ,x(j+(m-1)\tau )\}\\ \vdots \\ X(N-(m-1)\tau )=\{x(N-(m-1)\tau ),x(N-(m-2)\tau ),\ldots ,x(N)\} \end{array}\right]$$

where *m* is the embedding dimension and *τ* is the embedding delay time. The embedding dimension and embedding delay are free parameters tailored to the time series. A higher embedding dimension enables the detection of more complex patterns in the time series, but at the cost of computation time and statistical precision. The embedding delay defines the time scale for describing the complex structure and it is an integer multiple of the sampling period [32,33].

Each row in the matrix can be treated as a reconstructed component, with a total of N−(m−1)τ. Then the reconstructed component X(j) is ranked in ascending order according to the value size, that is $x[j+(j_{1}-1)\tau ]\leq x[j+(j_{2}-1)\tau ]\leq \ldots \leq x[j+(j_{m}-1)\tau ]$, where, $j_{1},j_{2},\ldots ,j_{m}$ represent the index number of the columns in which each element is located in the reconstructed component. If there is $x[k+(j_{p}-1)\tau ]=x[k+(j_{q}-1)\tau ]$, sort by the size of *j*. Therefore, for each row in the matrix *X*, a set of symbol sequences can be obtained, that is, ordinal pattern $S(g)=(j_{1},j_{2},\ldots ,j_{m})$, where g=1,2,…,l. So, there are l≤m! ways of arranging symbol sequences. And calculate the ordinal pattern probability distribution $P_{1},P_{2},\ldots ,P_{l}$, obviously, $\sum_{g=1}^{l}P_{g}=1$.

Afterward, the PE is just the Shannon entropy estimated by using this ordinal pattern probability distribution:

(10) $$H_{p}(m)=-\sum_{g=1}^{l}P_{g}\ln P_{g}$$

The maximum value ln(m!) of $H_{p}(m)$ is obtained when $P_{g}=1/m!$ [32]. For convenience, $H_{p}(m)$ is typically normalized with ln(m!), namely:

(11) $$H_{p}=H_{p}/\ln(m!)$$

Obviously, $H_{p}$ ranges between 0 and 1. The magnitude of $H_{p}$ represents the randomness degree of the time series. The smaller the value of $H_{p}$ is, the more inerratic the time series will be, otherwise, the more stochastic the time series will be. The change in $H_{p}$ reflects and amplifies the minute details of the time series.

### 2.3. TFPF

Gear system signals y(n) can be modeled as:

(12) y(n)=x(n)+r(n)

where, x(n) is the valid signal component, which is generally considered to be composed of a number of band-limited, non-stationary, deterministic components; r(n) is the additive random noise, and n is the sampling point.

Firstly, using frequency modulation, the noisy signal y(n) is transformed to the instantaneous frequency of a unit amplitude analytic signal, which can be defined as follows:

(13) $$z_{y}(n)=e^{j2\pi \rho \sum_{m=0}^{n}y(m)}$$

Here, ρ is similar to the frequency modulation index. According to the definition of instantaneous frequency, the noisy signal y(n) is converted into the instantaneous frequency of the analytic signal $z_{y}(n)$.

Then, the peak value of the Wigner-Ville distribution (WVD) of $z_{y}(n)$ is taken to estimate the effective signal x(n). The instantaneous frequency estimate is obtained by taking the maximum value of WVD according to the frequency variable:

(14) $${\hat{f}}_{z}(n)=\frac{\arg\max[PW_{z}(n,f)]}{\rho }$$

where, argmax[·] is the operator which takes the maximum value along the frequency direction, while $PW_{z}(n,f)$ represents the WVD of $z_{y}(n)$. The WVD with time-varying window h(m) is defined as:

(15) $$PW_{z}(n,f)=\sum_{m=-\infty }^{\infty }h(m)\bar{z_{y}}(n-m)z_{y}(n+m)e^{-j4\pi fm}$$

where, $\bar{z_{y}}$ is the conjugate operator to $z_{y}$. The length of the window function h(m) is a parameter which influences the tradeoff between random noise attenuation and signal preservation.

Therefore, the process of instantaneous frequency estimation will not be affected by the noise, if the noise satisfies certain conditions. That is, the estimated instantaneous frequency is an estimate of the effective signal:

(16) $$\hat{x}(n)={\hat{f}}_{zy}(n)$$

### 2.4. Steps of CEEMDAN-PE-TFPF

In order to combine the advantages, the hybrid noise reduction algorithm CEEMDAN-PE-TFPF is proposed. There are four steps to the proposed algorithm.

• Step 1: Decomposition.

The noisy signal is decomposed to obtain $IMF_{i}(i=1,2,\cdots ,I)$ using the CEEMDAN algorithm.

• Step 2: Classification.

In order to classify whether the IMFs should be filtered, the PE value of each IMF is calculated, and then the IMFs are classified according to their PE values. Experimental results have shown that the permutation entropy of the signal represents the randomness of the signal and that the larger the value is, the more random the signal is. Generally, if the PE of the signal is greater than a threshold *θ*, the signal is considered to be relatively noisy, otherwise it is approximately considered clean. The PE value threshold *θ* was chosen as a result of literature review and many experiments involving the estimation of the PE values of the simulation representative signals. Through trial and error, it is found that *θ* is taken as 0.6 is more appropriate.

• Step 3: De-noising.

The IMFs that do not require filtering are retained directly, while the other components are de-noised using different window lengths. Considering the characteristics of TFPF, a short-window TFPF used to preserve the valid component signal as much as possible, and long-window TFPF is used to reduce the random noise as much as possible.

• Step 4: Reconstruction.

**Figure 2:**
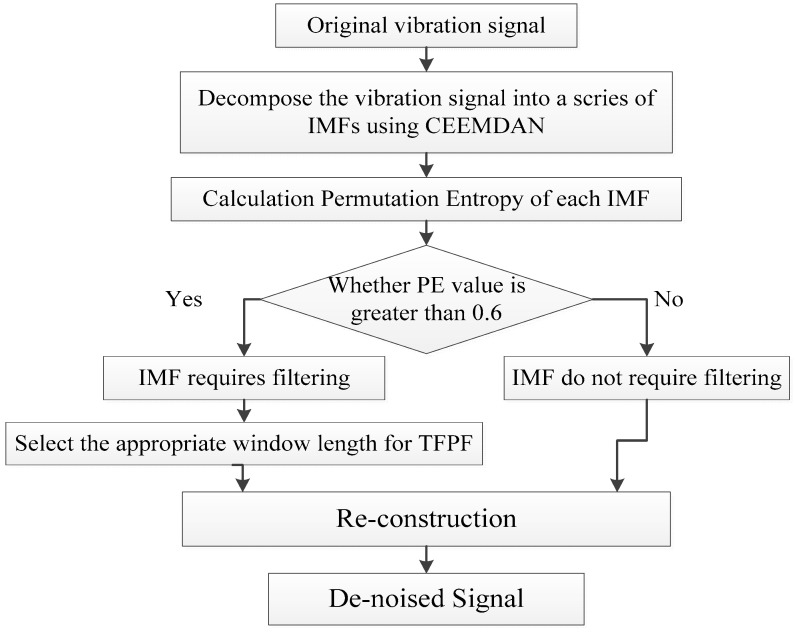
Framework of the proposed method.

The final filtered signal is obtained by reconstructing the de-noised IMFs and the retained IMFs. Figure 2 shows all the steps of the proposed CEEMDAN-PE-TFPF algorithm.

## 3. Application to Simulated Signals

**Figure 3:**
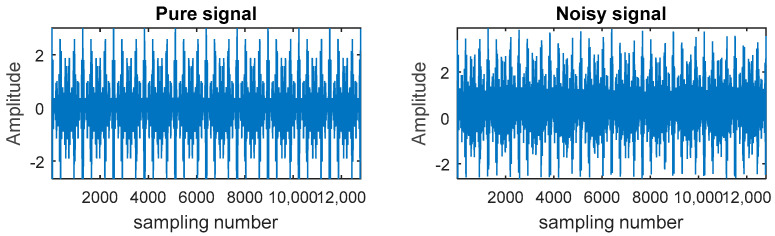
The pure signal and the noisy signal.

A simulated signal x(t), shown in Figure 3, was utilized to explain the principle of the CEEMDAN-PE-TFPF algorithm, where noise is the added Gaussian white noise. The modulation frequencies were $f_{m1}=40\;\mathrm{Hz}$ and $f_{m2}=70\;\mathrm{Hz}$, while the carrier frequency is $f_{c}=300\;\mathrm{Hz}$.

(17) $$x(t)=[1+\cos(2\pi f_{m1}t)+\cos(2\pi f_{m2}t)]\cdot \cos(2\pi f_{c}t)+noise$$

**Figure 4:**
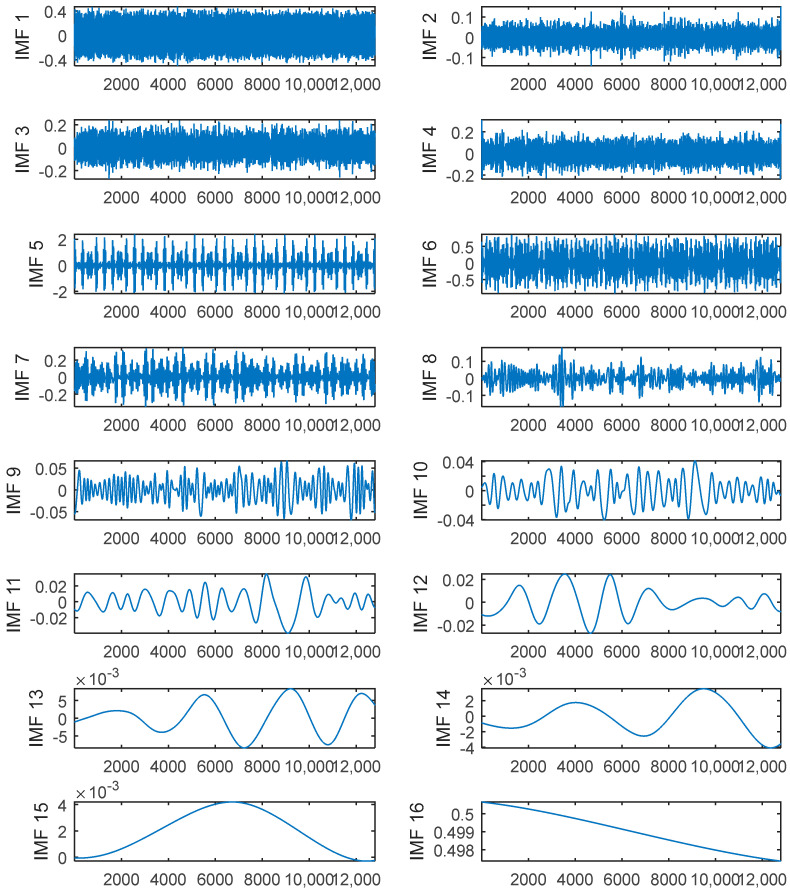
The IMF obtained by CEEMDAN algorithm.

Firstly, the signal was decomposed using the CEEMDAN, where the ratio of standard deviation of added white noise is 0.2 and the ensemble number is 500. Figure 4 shows that the noisy signal was decomposed into sixteen IMFs.

The PE value of each IMF was calculated and listed in Table 1. Then the IMFs were classified into those which should be filtered and those that should not according their PE value.

**Figure 5:**
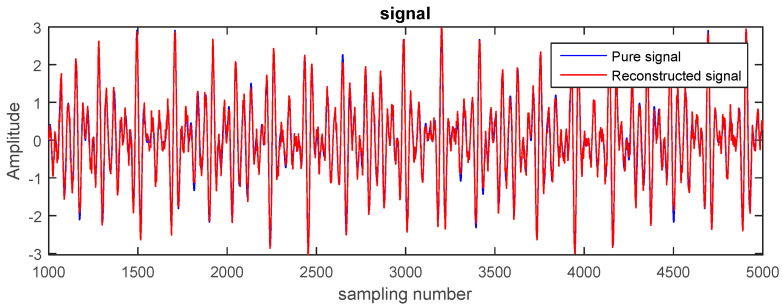
The reconstructed signals.

Therefore, as the PEs of the first, second, and third modes were larger than 0.6, these IMFs needed to be de-noised. The noise was reduced using TFPF with different window lengths according to the PE value. In this paper, the first two were filtered using a long window TFPF, and the third was filtered using a short window TFPF. The reconstructed signal shown in Figure 5 is the sum of the components of the de-noised and retained modes. By contrasting between the pure and reconstructed signals, the proposed method can be used to almost completely reconstruct the pure signal without causing a lot of loss of the effective signal and excessive residual noise.

To compare the reconstruction results, CEEMDAN-PE-TFPF, EMD-PE-TFPF, fixed-window TFPF, and the wavelet transform (with a mother wavelet of db4, and five levels of decomposition) were used to reconstruct the signal. The output SNR and MSE [34] were calculated to evaluate the reconstructed result:

(18) $$SNR=10\log_{10}(\frac{\sum_{n=1}^{N}{\left(y(n)\right)}^{2}}{\sum_{n=1}^{N}{\left(y(n)-\bar{y}(n)\right)}^{2}})$$

(19) $$MSE=\frac{1}{N}\sum_{n=1}^{N}{\left(y(n)-\bar{y}(n)\right)}^{2}$$

where, y(n) is the noisy signal, and $\bar{y}(n)$ is the reconstructed signal. Table 2 shows the SNR and MSE values obtained using different de-noising methods. Based on the results, we conclude that the SNR of the signal reconstructed using the CEEMDAN-PE-TFPF is larger than that of others, while its MSE is smaller. These results show that the proposed reconstruction method is superior to other methods.

**Figure 6:**
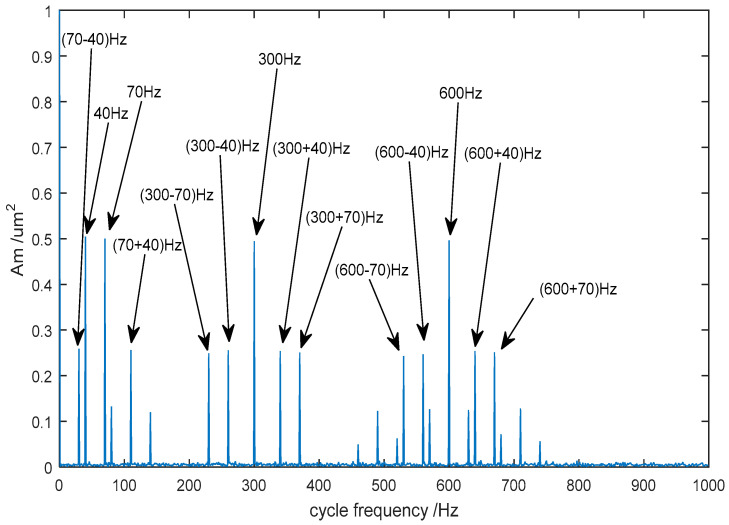
The cyclic autocorrelation spectrum of the de-noised signal.

When the gear transmission system is subjected to faults, the frequency components and amplitude of the vibration signal will change, so an amplitude modulation effect and a frequency modulation effect occur simultaneously. In order to extract the fault’s frequency characteristic from the vibration signal, it is necessary to demodulate and analyze the de-noised signal. As shown in Figure 6, 40 Hz and 70 Hz components appear at low frequencies, which is the modulation frequency of the original signal. The carrier frequency (300 Hz) and its double frequency (600 Hz) are obviously prominent at high frequencies, and the edge frequency band characteristic separated by the 40 Hz and 70 Hz is also demodulated clearly. Therefore, the de-noised method of CEEMDAN-PE-TFPF not only retains the useful signal, but also removes the noise to the maximum extent.

## 4. Experimental Verification

In this section, in order to further demonstrate the effectiveness of the proposed method, an experiment on a test bench of gear transmission system is presented, where the vibration signal is extracted.

**Figure 7:**
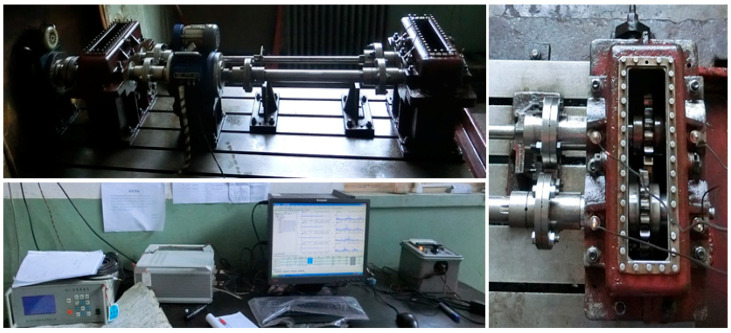
Experimental setup for signal acquisition of the gear transmission system.

As shown in Figure 7, the experimental setup mainly consisted of a main test gearbox, an accompanying test gearbox, accelerometers, speed and torque meters and a torsion bar. The four accelerometers were installed on the bearing base of the driving and driven gears. During the experiment, the whole driving system was driven by the motor, and the torque were measured from the torque meter by the torsion bar. The parameters of the single-stage spur gearbox are shown in Table 3. A dynamic data acquisition and analysis system was used to collect the vibration signal, and the de-noising process is the same as previously presented, comprising decomposition, classification, de-noising and reconstruction.

**Figure 8:**
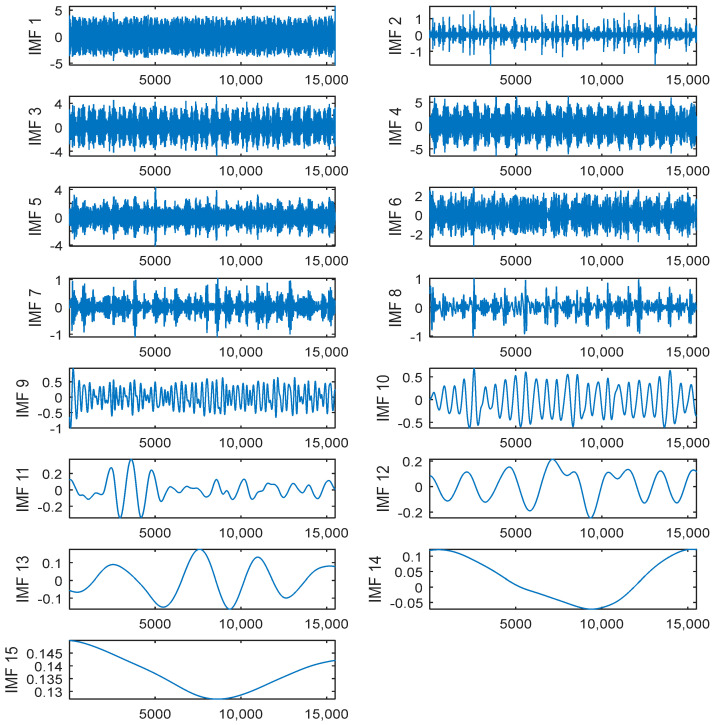
The IMFs obtained by the CEEMDAN algorithm.

**Figure 9:**
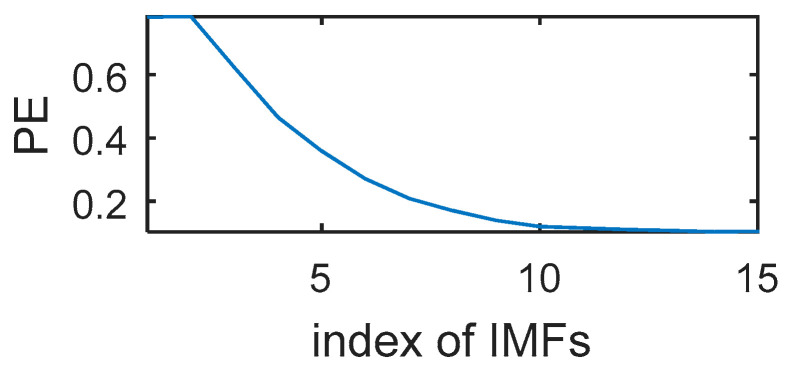
The PE values of IMFs.

**Figure 10:**
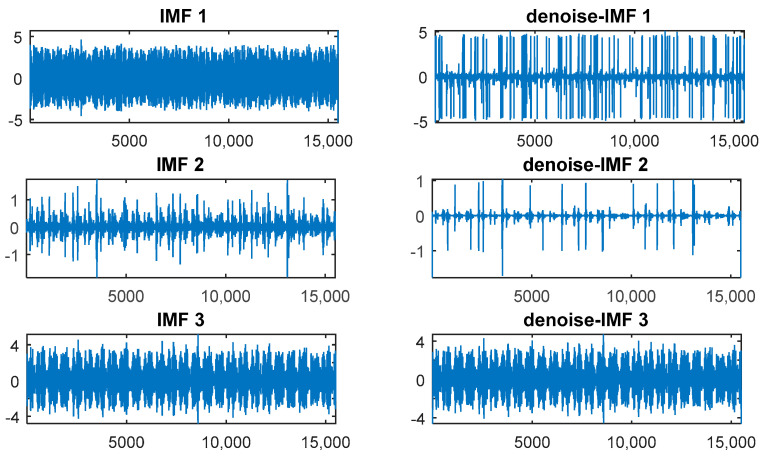
De-noising results of the IMFs.

**Figure 11:**
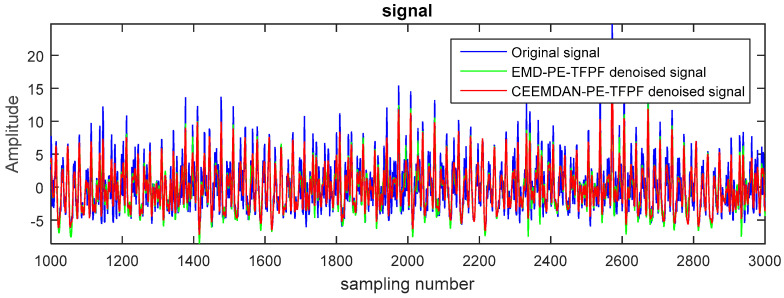
The de-noised signal by CEEMDAN-PE-TFPF and EMD-PE-TFPF.

**Figure 12:**
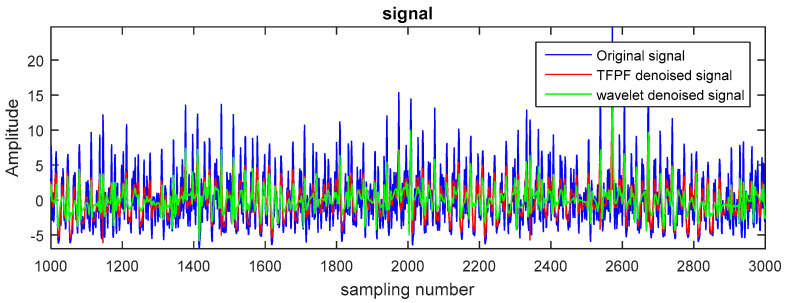
The de-noised signal by TFPF and wavelet.

Figure 8 shows the 15 IMFs obtained and decomposed using CEEMDAN. Then according to their corresponding PE values (Figure 9), the first two IMFs were filtered using long window TFPF, and the third IMF was filtered using short window TFPF. Figure 10 shows that the random noise is effectively suppressed through the long-window TFPF, and the valid signal amplitude is preserved through the short-window TFPF. Finally, all the IMF components were used to reconstruct the de-noised signal, shown in Figure 11. To illustrate the superiority of the CEEMDAN-PE-TFPF method, the EMD-PE-TFPF, TFPF, and the wavelet transform were also applied. Figure 11 is the result of the EMD-PE-TFPF method, where it can be seen that noise is not suppressed effectively, whereas in Figure 12 the results of the TFPF and wavelet transform are shown, where it is clear that a portion of the signal is also removed.

Furthermore, the SNR and MSE were calculated to evaluate the reconstructed result quantitatively. As shown in Table 4, the method of CEEMDAN is more effective than EMD in obtaining more accurate IMFs. Therefore, we can see that after wavelet denoising, the randomness of the signal still exists and it is obvious that the de-noised result of TFPF is superior to that of the wavelet method. It is also evident that traditional TFPF after signal decomposition contributes to the noise reduction performance of the algorithm. What’s more, the proposed CEEMDAN-PE-TFPF can reduce the noise present effectively and at the same time preserve the valid signal.

**Figure 13:**
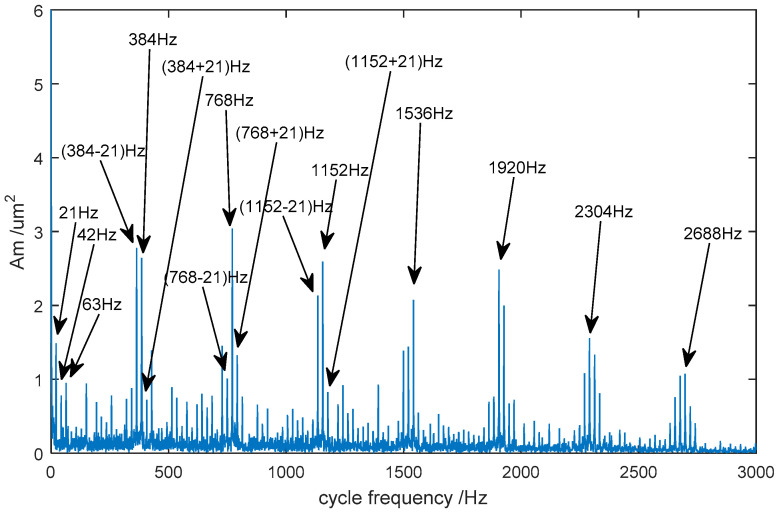
The cyclic autocorrelation spectrum of the de-noised signal.

In order to extract the fault’s frequency characteristic, the signal de-noised using the proposed CEEMDAN-PE-TFPF method was analyzed using the cyclic autocorrelation spectrum. The result is shown in Figure 13.

**Figure 14:**
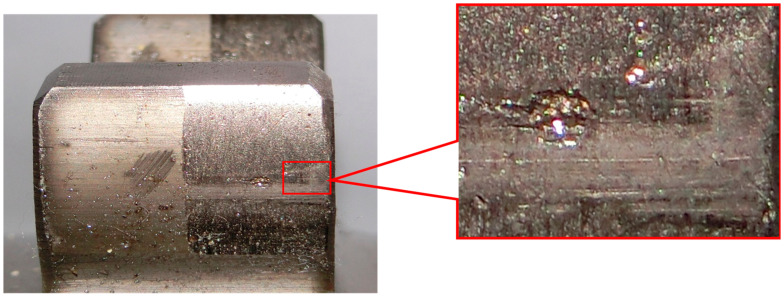
Gear tooth pitting.

In this case, the rotation rate was 1280 r/min, the meshing frequency of the gear was 384 Hz and the rotation frequency of the gear was 21 Hz. It can be seen from the figure that the amplitude of the carrier frequency at 384 Hz and its harmonic frequencies are larger. What’s more, a series of edge frequency bands, separated by the rotation frequency of 21 Hz, are concentrated on both sides of the meshing and harmonic frequencies. Therefore, it can be concluded that the gear system has broken down and the fault is likely to be pitting. Owing to pitting that occurs in the gears, the frequency components of the vibration waveform will contain a carrier frequency and a harmonic frequency. Furthermore, the edge frequency band is distributed at intervals of the rotation frequency on both sides of the carrier frequency and harmonic frequency. From Figure 14, it can be seen that the gear has suffered pitting.

## 5. Conclusions

This paper proposed a hybrid algorithm that combines TFPF with the CEEMDAN and PE methods for reducing noise in a gear transmission system’s vibration signal. By utilizing the decomposition characteristics of CEEMDAN and the value of PE, the window length of TFPF is chosen adaptively. Results demonstrate that the proposed method achieves a good tradeoff between noise suppression and signal preservation, especially compared with EMD-PE-TFPF, traditional TFPF, and wavelet transform.

The purpose of this paper is to verify the de-noising performance of the proposed method. The effectiveness of the CEEMDAN-PE-TFPF algorithm was also verified by calculating the values of the SNR and MSE, and the fault characteristics of the gear system were extracted effectively from the de-noised signal after analyzing the cyclic autocorrelation spectrum. Additionally, the proposed CEEMDAN-PE-TFPF de-noising algorithm can also be applied to other systems for de-noising. In our next work, artificial intelligence techniques will be employed in combination with the CEEMDAN-PE-TFPF algorithm for accurate signal processing and fault diagnosis.

## Figures and Tables

**Table 1:** The PE values of IMFs.

IMF	PE	IMF	PE	IMF	PE	IMF	PE
IMF1	0.9178	IMF5	0.3174	IMF9	0.1472	IMF13	0.1085
IMF2	0.8423	IMF6	0.2567	IMF10	0.1263	IMF14	0.0964
IMF3	0.7465	IMF7	0.2125	IMF11	0.1165	IMF15	0.1044
IMF4	0.5745	IMF8	0.1772	IMF12	0.1109	IMF16	0

**Table 2:** Value of SNR and MSE of different reconstructed signal methods.

Methods	Original	CEEMDAN-PE-TFPF	EMD-PE-TFPF	TFPF	Wavelet
SNR (dB)	13.9039	32.6742	30.1352	28.1960	17.3368
MSE	0.3324	0.0509	0.0656	0.0796	0.2358

**Table 3:** Parameters of the single-stage spur gearbox.

Parameters	Values/Description
tooth number	18
module	8 mm
pressure angle	20°
tooth width	10 mm
gear material	42 CrMn
torque	837 Nm
rotation rate	1280 r/min
sampling frequency	12,800 Hz

**Table 4:** Value of SNR and MSE of different reconstructed signal methods.

Methods	CEEMDAN-PE-TFPF	EMD-PE-TFPF	TFPF	Wavelet
SNR (dB)	16.7115	14.7314	7.3790	5.2352
MSE	2.9332	3.5754	7.4582	9.2415
